# Machine learning and image-based profiling in drug discovery

**DOI:** 10.1016/j.coisb.2018.05.004

**Published:** 2018-08

**Authors:** Christian Scheeder, Florian Heigwer, Michael Boutros

**Affiliations:** German Cancer Research Center (DKFZ), Division Signaling and Functional Genomics and Heidelberg University, Department of Cell and Molecular Biology, Medical Faculty Mannheim, D-69120 Heidelberg, Germany

**Keywords:** Imaging, Image analysis, Machine learning, Drug discovery, High-throughput screening, High-content analysis

## Abstract

The increase in imaging throughput, new analytical frameworks and high-performance computational resources open new avenues for data-rich phenotypic profiling of small molecules in drug discovery. Image-based profiling assays assessing single-cell phenotypes have been used to explore mechanisms of action, target efficacy and toxicity of small molecules. Technological advances to generate large data sets together with new machine learning approaches for the analysis of high-dimensional profiling data create opportunities to improve many steps in drug discovery. In this review, we will discuss how recent studies applied machine learning approaches in functional profiling workflows with a focus on chemical genetics. While their utility in image-based screening and profiling is predictably evident, examples of novel insights beyond the status quo based on the applications of machine learning approaches are just beginning to emerge. To enable discoveries, future studies also need to develop methodologies that lower the entry barriers to high-throughput profiling experiments by streamlining image-based profiling assays and providing applications for advanced learning technologies such as easy to deploy deep neural networks.

## Introduction

Using complex phenotypes to predict functions of genes has been a powerful approach in genetics and clustering mutations by their organismal phenotypes has yielded important insights into functions of many genes and pathways [1]. Visual phenotypes in genetic screens were usually manually scored and phenotypes clustered by similarity. These approaches yielded fundamental insights into the components and architecture of many conserved signaling pathways that have later been found to be recurrently mutated in many diseases 2, 3, 4.

More recently, automated microscopy-based phenotyping has become a powerful method to infer functions and functional relationships of genes, to investigate cellular or organismal structure, behavior and disease mechanisms 5, 6, 7. Automated imaging-based methods comprise a broad range of qualitative and quantitative strategies to measure phenotypes in various systems ranging from unicellular organisms to cell lines and whole animals 3, 8, ∗∗9, 10, 11, 12, however, challenges are often similar, from standardization of imaging conditions to an automated approach to analyze very large data sets.

In drug discovery, automated imaging was used in a number of different applications in pre-clinical development and has been shown to allow scalable and systematic phenotypic profiling of small molecules, establishing itself as a complementary method to target-based in-vitro screening 7, 13, 14. In this review, we will summarize the current state of the art with a focus on recent publications that highlight novel analysis methodologies for image-based screening. While most methods are applicable in any perturbation-based screens, this review in particular highlights applications in chemical genetics and drug discovery.

### Image-based profiling in drug discovery

For image-based phenotyping of perturbations, one can distinguish two approaches to experimental design that fundamentally differ with regards to probes as well as image analysis 10, ∗15, 16.

**Figure 1:**
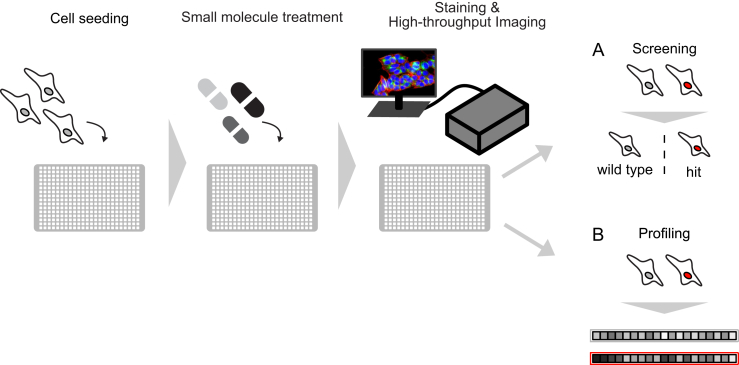
Typical workflow of image-based small molecule experiments. A typical high-throughput imaging experiment starts with the seeding of adherent cells in suitable microtiter plates (e.g. 384-well plates) followed by an incubation time of several hours to let the cells attach. In a second step, compounds are added using robotic liquid handling stations. In simple experiments cells are perturbed with one compound per well before the assay is stopped by fixation and staining of cells. Finally, plates are imaged using automated microscopes (wide-field or confocal, one or more fields on view, [23]). This generic workflow describes the basic steps of high-throughput imaging experiments which can further be distinguished in two approaches. (A) Screening experiments are designed to identify small molecules that modulate a pre-defined phenotype. (B) Profiling experiments follow an unbiased approach to profile cells upon perturbations by extracting hundreds of phenotypic measurements. In both approaches the application of more complex experimental designs and cell culture models such as 3-D cell culture will require specific microscopes, additional processing steps and, thus, reduce throughput.

The first approach includes screening applications (often called high-content or phenotypic screening) that are focused on pre-defined, specific phenotypes with the aim to identify drugs or drug targets that modulate it (Figure 1A). Such image-based phenotypic screens have, for example, been successfully used to identify targets and compounds that modulate phenotypes like the subcellular localization of specific proteins 17, 18.

**Figure 2:**
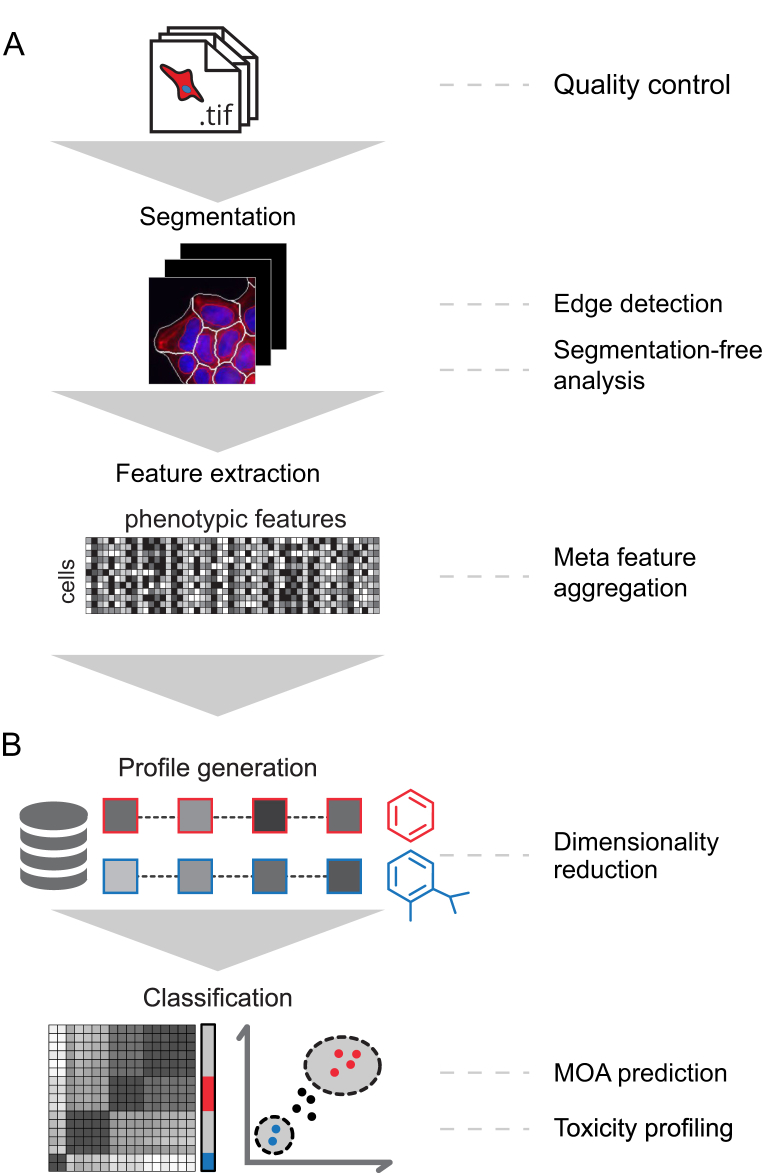
Typical analysis strategy and machine learning applications in image-based small molecule profiling experiments. (A) Images acquired in a high-throughput profiling experiment are analyzed using automated, highly parallelized analysis pipelines that employ software such as CellProfiler, R/EBImage, Icy or ImageJ. In a first step, the quality of the images is controlled. For this purpose, machine learning classifiers can be trained to recognize and remove images with artifacts. Segmentation-based or segmentation-free approaches are then used to extract quantitative image features that represent the cellular phenotypes (see Box 1 for more details). (B) The extracted phenotypic features may be used “as is” or further processed to derive meta-feature vectors representing each perturbation. Treatment-level profiles are then further used to classify compounds with shared MOA, toxicity or to assess the efficacy of candidate molecules.

The second application of high-throughput imaging in drug discovery is the more global profiling of perturbations. This approach profiles cells upon exposure to genetic, pathogenic or chemical perturbations and is complementary to techniques like transcriptional profiling (Figure 1B, 19, 20, 21). Subcellular structures are stained with multiplexed fluorescent dyes and fluorescently labeled antibodies to visualize or ‘paint’ cells and subcellular structures. Automated image acquisition and analysis are subsequently used to profile phenotypes of cells in an unbiased manner (Figure 2, Box 1, 22, ∗23). Computer vision can extract multivariate feature vectors of cell morphology such as cell size, shape, texture and staining intensity without further human intervention (Figure 2A). All large-scale studies reported so far employed segmentation approaches to accurately define cellular outlines prior to feature extraction. The derived profiles of single cells or cell populations are then scored to find relationships within data sets comprising up to tens of thousands of perturbations (Figure 2B, 24, ∗∗25, 26).

Box 1 **Processes in image analysis workflows and machine learning applications.**

**Segmentation**

High-throughput small molecule profiling is typically based on the staining of subcellular structures and fluorescence microscopy. A selective staining of abundant subcellular structures such as DNA or actin and the subsequent imaging in distinct channels allows the identification of single cells as objects [23]. An accurate segmentation of nuclei and cell bodies can be achieved by intensity-based thresholding and region growing approaches 81, 82, 83. For more complex cell shapes or multicellular objects such as organoids, more sophisticated models are required to segment objects of interest [84]. A number of computational applications for segmentation-free analysis in image-based profiling have been developed 85, 86. Recently, deep learning approaches based on segmentation-free strategies, in some cases in combination with single-cell segmentation, have been developed for image-based small molecule profiling (see main text for further details).

**Feature extraction**

Following segmentation, computer vision is used to extract morphology, intensity and texture features of single cells 81, 82. Morphological features describing cellular size and shape are calculated based on the cell outlines. Intensity and texture features are calculated on pixel values of raw or pre-processed images within those outlines. These features are defined based on human knowledge. Hundreds to thousands of features are extracted to achieve an unbiased character of the studies. In contrast, segmentation-free approaches provide a hypothesis-free approach for feature extraction independent of segmentation outlines. Recently, several approaches that use deep neural networks for segmentation-free feature extraction were developed 72, 73, 74, 75. While the features extracted by deep neural networks cannot be readily interpreted by humans, results from pilot studies indicate that those features are complementary to those extracted by segmentation-based strategies [73].

**Profile generation**

Segmentation-based image analysis results in collections of single cell feature vectors for each condition (e.g. per small molecule treatment). Various strategies have been explored to process those single cell feature vectors into profiles for downstream analyses. A common method relies on the aggregation of single cell features by summarizing the mean and standard deviation over all cells of a perturbation and other methods using machine learning approaches to generate profiles are reported [60]. Furthermore, features are often highly correlated and deliver very similar information content. This inflates data complexity and impairs downstream analysis. Strategies to either select features carrying redundant information or to reduce data dimensionality by transformations are thus commonly applied before proceeding with downstream analyses [59].

**Classification**

The aim of many profiling experiments is a classification of compounds. Based on a set of reference compounds and their profiles, classification strategies are used to infer putative MOA or toxicity relationships of formerly unseen compounds. This process is a bona fide machine learning task. Approaches include hierarchical clustering of compounds into groups that share high profile similarity, training support vector machines on profiles for MOA prediction or correlation network analysis ∗∗59, ∗60, 61. More recently, advanced classifiers such as random forests or deep neural networks were employed in order to achieve higher accuracy in predicting MOAs 71, 74, 75. The latter however with an increased complexity for analysis and demand more in-depth knowledge of neural network-based machine learning.

Alt-text: Box 1

**Figure 3:**
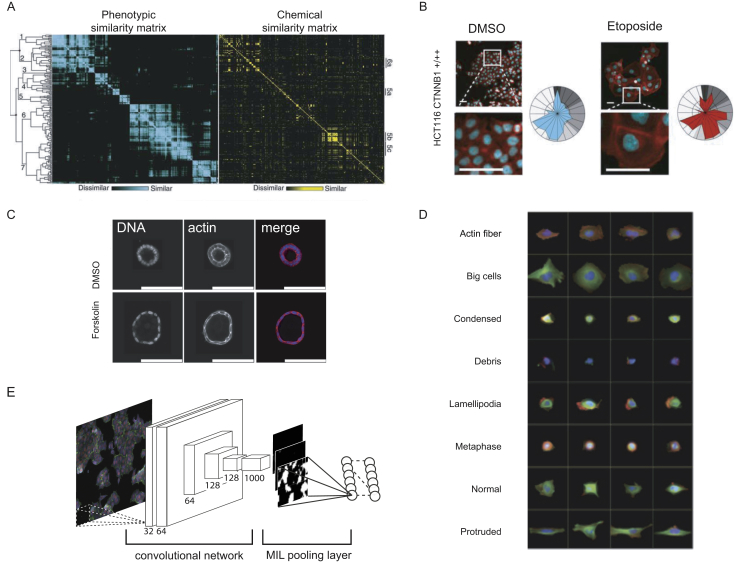
Example images and analyses for image-based small molecule profiling. (A) Young et al. could show that substantial correlation between phenotypic feature vectors and chemical similarities based on compound chemical structure exists. This implied that phenotypic similarities could be harnessed to find small molecules with similar chemical structure (adapted from Ref. [28]). (B) Breinig et al. built on this observation and tested if image-based phenotypic profiles also reveal cell line specific responses to certain compound perturbations. They found, for example, that the presence or absence of the CTNNB1 mutant allele causes a differential cellular reaction to Etoposide treatment (adapted from Ref. [25]). (C) 3D-cyst culture was applied by Booij et al. to map the activity and function of small molecule compounds and antibodies by image-based profiling. Using this analysis, they found that PI3K inhibitor treatment was not able to suppress forskolin induced cyst swelling (adapted from Ref. [68]). (D) Schematic illustration of the neural network architecture (left) that was used by Kraus et al. to classify small molecules by mechanism of action from full-size microscopy images of cancer cells. Kraus et al. combined deep convolutional networks with multiple instance learning (MIL) to classify small molecules based on image-level labels. In a second step the architecture of the convolutional MIL model allowed tracing back and segmenting single cells in the full-size images ([71], figure kindly provided by O. Kraus). (E) Fuchs et al. employed a classifier to group cells into phenotypic classes. By classifying single cells phenotypic class upon RNAi perturbation they could group genes based on phenotypic similarity and functionally related groups (adapted from Ref. [16]).

High-throughput imaging has been successfully used for small molecule profiling in human cancer cells to group small molecules with similar activity and mechanism of action (MOA) [27]. The concept of clustering small molecules based on the similarity of phenotypic profiles was further exploited to correlate phenotypic responses with chemical structure similarity (Figure 3A and B, ∗28, 29). The basic principle of grouping small molecules based on similarity of measured phenotypic profiles has further been successfully applied in a variety of drug discovery applications. For example, annotated libraries of pharmacologically active small molecules could be profiled to hypothesize off-targets and to suggest drug-target pairs with possibilities for repurposing ∗∗25, 30. Chemical genetics approaches also allow a systematic survey for drug repurposing by linking the effect of small molecules with the absence or presence of loss- or gain-of-function alleles ∗∗25, 31. Further strategies include the association of small molecules to a new target by comparing its induced phenotype to that of a reference molecule and the unbiased testing of small molecules for candidates that reverse a disease-associated cellular phenotype to the wild-type phenotype ∗∗25, 30, 32. Other applications include MOA profiling of natural product libraries and the enrichment of large compound libraries for effective and diverse subsets 33, 34. Studies also demonstrated the application of image-based profiling for toxicity profiling, for example, by using model systems such as 3-D liver cell culture or induced stem cell-derived cardiomyocytes to assess small molecule toxicity 35, 36. Additional applications include the unbiased identification of toxic small molecules in large natural product libraries 33, 37, 38. A further optimization of image-based profiling assays and analysis concepts made the technology accessible to more complex drug discovery studies as demonstrated by a recent study in which 50,000 compounds were profiled in pluripotent stem cells to complement a targeted antibody readout [39].

Image-based phenotypic profiling has also been recently applied to characterize small molecule function in patient-derived primary cells or 3-D cell culture models. A recent study by Booij et al. used image-based profiling to quantify the phenotypic effects of kinase inhibitors in a 3-D cell culture model of Polycystic kidney disease to facilitate novel hypotheses for small molecule treatments (Figure 3C, [40]). Another study deployed image-based profiling in primary chronic lymphatic leukemia (CLL) cells to identify small molecules effective in overcoming resistance against the BCL2 inhibitor Venetoclax [41].

### Chemical genetics and image-based profiling

Chemical genetics approaches have been pioneered in model organisms to discover drug MOA as well as gene functions 42, 43, 44, 45, 46. In such screens, libraries of loss-of-function or gain-of-function mutant strains were screened against chemical libraries to measure genotype-dependent changes in fitness. Comparisons of phenotypic responses of mutant strains across chemical perturbations allowed to map genes with shared function and, by assessing the response across a panel of mutants, the bioactivity and cellular targets of small molecules [31].

In model organisms like yeast or bacteria, sophisticated experimental and analysis frameworks have been established for chemical-genetic studies which might provide further strategies for profiling in human cells 31, 47. As an example, a recent study in *Saccharomyces cerevisiae* used an algorithm to define a set of query mutant strains from genetic-interaction data. Several chemical libraries were then screened against the selection of deletion strains with the aim to functionally annotate small molecules across relevant biological processes without screening the full deletion strain library [48].

This principle has been adopted to pharmacogenomics studies in human cancer cell lines by integrating drug response and the genetic background of cell lines with the aim to identify cancer-specific vulnerabilities 49, 50, 51. Several large-scale pharmacogenomics studies relied on univariate phenotypic readouts based on cell viability assays that can be applied to a large panel of cell lines, however, not all univariate cell variability measurements are equivalent 49, 50, 52.

Microscopy-based profiling experiments are typically carried out in microtiter plates, which enables a rapid scaling of experiments for a large number of perturbations, *i.e.* to profile chemical libraries in a large number of genetic perturbations. As such, image-based chemical genetics offers a great potential to systematically characterize small molecules in human cell-based systems and to probe chemical-genetic interactions in complex and monogenic human diseases 7, 53. To systematically map chemical-genetic interactions by image-based profiling, smaller panels of human cancer cell lines have been used (Figure 3B, ∗∗25, 54, 55. The unbiased measurement of chemical genetic interactions across a wide phenotypic space can identify chemical-genetic interactions, small molecule off-target effects and drug repurposing opportunities. The establishment of image-based assays is often more labor intensive and not every cell line is suitable for high-throughput image-based profiling experiments [23].

### Machine learning strategies for image-based profiling

High-throughput microscopy generates large collections of phenotypic data. The size, complexity and multiparametric nature of such data sets make automated analysis strategies necessary to identify cellular phenotypes that differ from wild type phenotypes and discover novel relationships between phenotypes and the perturbagens which induce them. Simple statistical inference methods such as mean values are often insufficient to comprehensively describe the complexity of data sets [56]. As a consequence, many profiling studies use supervised or unsupervised machine learning strategies to capture and evaluate phenotypic changes of single cells or populations of cells (Box 2, Figure 3D, 57, 58, ∗∗59).

Box 2 **Basic principles of machine learning.**

Machine learning strategies can generally be separated in unsupervised and supervised approaches.

**Unsupervised machine learning** approaches interpret and learn an abstract representation of the intrinsic structure of data sets and can be applied without *a priori* definition of labels (phenotypes) to be classified. A commonly applied unsupervised machine learning strategy is clustering of data points into patterns to ultimately derive biologically meaningful information ∗27, ∗28, ∗60. Commonly applied unsupervised clustering algorithms include hierarchical or k-means clustering.

By contrast, **supervised machine learning** strategies are typically applied to classification problems and thus rely on pre-defined classes. Annotated training sets with data points representing those classes are used to train a classifier 16, 65. This way, the classifier learns how to distinguish between the classes based on the measured variables (also called **features**). Linear and non-linear supervised machine learning classifiers exist, and the choice of the classifiers depends on the classification problem. In many cases, a comparison of the classifier performance is used to identify the algorithm that is best suited for the problem. The training of classifiers requires representative training data sets of sufficient size and quality. To avoid issues such as overfitting and assess classification accuracy statistics, the resulting classifier needs to be evaluated carefully using test data (not used for training).

Recently, **deep learning** has been exploited as a supervised machine learning technology for biological classification problems. Deep learning uses artificial neural networks, which consist of multiple layers of interconnected linear or non-linear transformations that are applied to the data with the goal to solve a classification problem. The networks are trained over multiple epochs, each time comparing the predicted and true class labels to optimize network parameters with automated algorithms [87]. The actual network architecture relies on complex mathematical concepts. Strategies such as **transfer learning** or **generic deep neural networks** provide possibilities for non-experts to apply deep learning without full knowledge of the mathematical details ∗∗9, 70, 73.

The high dimensionality and complexity of image data sets typically make analysis strategies with multiple steps and combinations of machine learning algorithms necessary. Here, deep learning strategies provide great potential to automate analysis at many stages from segmentation, feature extraction to classification to reduce manual tuning of analysis pipelines 71, 74.

Alt-text: Box 2

Clustering image-based profiles to identify small molecules with shared MAO based on the ‘guilt-by-association’ rule relies on unsupervised machine learning strategies ∗∗25, ∗27, ∗28, 34. In the simplest case, morphological profiles are generated by aggregating all single-cell measurements per treatment condition and several statistical methods have been applied to generate morphological profiles. When used to classify a set of compounds by their MOA, a comparison of profiling methods showed only minimal differences between them [60].

For large data sets, hierarchical (unsupervised) clustering algorithms are commonly used to cluster small molecule profiles based on their profile similarity. Often, matrices of all pairwise similarities between small molecule profiles are used to derive a distance measure (*i.e.* 1 – similarity) rather than absolute distance measures such as the Euclidian distance [61]. Furthermore, phenotypic similarity matrices correlate in some cases with other similarities of small molecules such as chemical similarity ∗∗25, ∗28.

Hierarchal clustering and the corresponding visualization as heatmaps are relatively easy to implement and require only few computational resources, even for large data sets. Furthermore, the concept of similarity-based clustering is frequently applied in other fields such as gene expression profiling or genetic interaction studies making those analyses and visualizations accessible to a broad audience 62, 63. Depending on the size and characteristics of the data set, alternative cluster algorithms and visualizations as for example two-dimensional maps might be favorable to identify relevant groups of cells or perturbations 16, 30, 64.

In contrast to unsupervised approaches, supervised machine learning algorithms are commonly applied when cells or populations of cells need to be classified in discrete phenotypic classes. Supervised machine learning has been successfully used within image-based genetic screening experiments to classify single cells into pre-defined, biologically meaningful classes based on their phenotypic profiles (Figure 3D, 16, 64, 65). Supervised classifiers are trained to learn the relevant phenotypes from training data to distinguish different phenotypes. This approach has been applied by using tool compounds, genetic perturbation or extracellular stimuli to create reference profiles which were further used to train classifiers 32, ∗39, 41, 66, 67, 68. In some cases, supervised machine learning strategies were used to calculate profiles of meta features which were then applied to various downstream analysis strategies [60]. As an example, Loo et al. developed a strategy using support vector machines to separate treated from untreated cells in the high-dimensional feature space. In subsequent steps, profiles were calculated using classification accuracy and the orientation of support-vector hyperplanes. The derived meta-profiles incorporate multiphasic responses over dose ranges and retain human interpretable profiles for analyses [69].

Supervised machine learning requires curated training data, e.g. manually labeled cells or defined experimental measurements. The size and quality of the training data is crucial for the performance of the corresponding classifier and manual annotation of samples may be biased by subjective decisions. As for many published studies that used supervised machine learning, training data sets need to be reviewed and possibly re-generated if only small experimental parameters change 57, 58. Thus, the choice of the machine learning algorithm and its proper implementation are key factors to avoid issues such as overfitting. Whilst such challenges might put unsupervised and simple statistical inference methods in favor for large-scale profiling experiments, supervised machine learning is being used with success to classify complex phenotypes 10, 16, 65.

A recent study that used high-throughput microscopy and automated image analysis with deep convolutional neural networks classified proteins by their subcellular localization in *S. cerevisiae* and thereby demonstrated the power of supervised machine learning for image-based phenotyping (Figure 3E, [9]). The deep neural network that was used not only outperformed a previously implemented ensemble of support vector machines at a complex automated image analysis task, but could also be adapted to new, divergent data sets using transfer learning.

Transfer learning aims to adopt a machine learning classifier to a new problem (i.e. new data sets, generated with e.g. different experimental parameters) by fine-tuning the classifier with training data from unseen data sets. Examples show that better classification performance can be achieved by combining deep- and transfer learning when compared to the setup of a classifiers trained from scratch ∗∗9, 70. This saves computational resources as the fine-tuning is computationally less expensive than training a deep neural network from scratch and high classification accuracy might be achieved with smaller training data sets.

Recently, a number of studies reported the application of supervised machine learning approaches with deep neural networks to classify small molecules by their MOA using annotated image data sets from the Broad Bioimage Benchmark Collection 70, 71, 72, 73, 74, 75. Various strategies with applications of deep neural networks at the level of feature extractions and/or classifications were reported, in some cases with previous traditional single cell segmentation. As an example, one study used labeled full resolution images to train a deep neural network which gave slightly better treatment level classification results compared to previously reported predictions using segmentation and factor analysis [71]. Notably, a relatively low number of images (25 images per treatment) were used to train the network and no previous segmentation and labeling of single cells was required.

Nevertheless, labeled training data sets imply the *a priori* knowledge of phenotypes, which can contradict the unbiased strategy of image-based profiling. Two recent studies propose to use generic deep neural networks that were pre-trained on millions of ‘consumer’ images for image-based profiling tasks 73, 75. The approaches are based on the assumption that generic neural networks learned general properties of natural images and are thus capable of extracting biologically meaningful information without additional training. Both studies report better results compared to traditional feature extraction when predicting small molecule MOA and provide a proof-of-concept for the applicability of generic deep neural networks for image-based small molecule profiling. As noted by the authors, additional studies with larger data sets across conditions to sample broader biological and technical space will be required for further validation.

Another recently explored application of supervised learning in image-based profiling, particularly deep neural networks, is a novelty detection framework to identify unexpected phenotypes [76]. Label-free profiling and the prediction of targeted drug screening assays are also future approaches exploiting image-based profiling data 77, 78, 79.

## Conclusions

Image-based profiling studies demonstrated the capability to improve the pre-clinical development of small molecules at almost any step of the pipeline from target identification over mechanism of action prediction to toxicity profiling. Increasing the throughput and extending more complex analysis methods of image based phenotypic screens and profiling approaches will help to increase the methodological portfolio of cellular screens to support the drug development process. Community efforts to create annotated datasets that can be shared across laboratories will be required to test and optimize the potential of strategies such as transfer learning to improve discovery science ∗∗59, 80.

Furthermore, large-scale chemical-genetics approaches inspired from successful studies in model organisms might harbor great potential to characterize drugs and drug-gene interactions in a systematic manner. Particularly, image-based profiling approaches in pre-selected informer panels of human cell lines might be a scalable and versatile tool to deprioritize compounds harboring adverse effects, asses compound efficacy and to generate hypotheses for drug synergism and repurposing.

## Funding

This work was in part supported by an ERC Advanced Grant (SYNGENE) of the European Commission.

## Conflicts of interest

None.
